# Supplementary figures and images for: Pre-exposure to mechanical ventilation and endotoxemia increases Pseudomonas aeruginosa growth in lung tissue during experimental porcine pneumonia
Source: PLoS One. 2020 Oct 27;15(10):e0240753. doi: 10.1371/journal.pone.0240753 (PMC7591049; doi:10.1371/journal.pone.0240753)

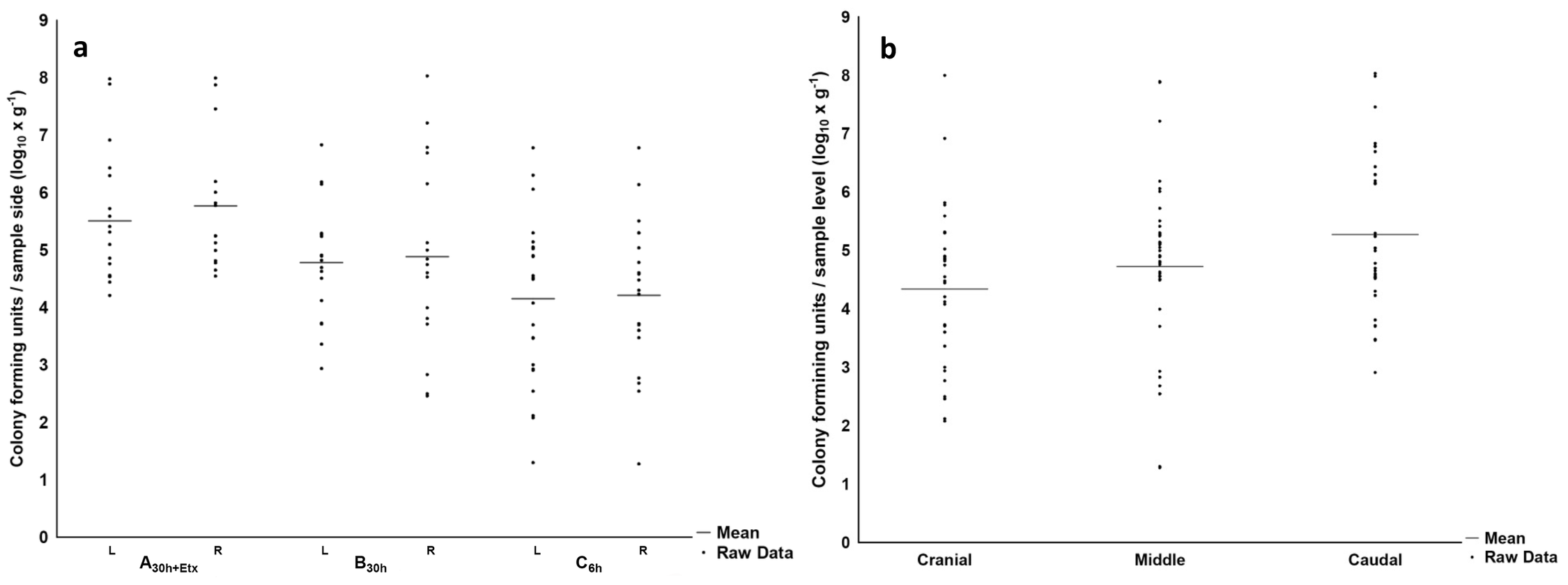

Supplement: S1 Fig — Raw data presentations of bacterial cultures in the experiment based on a) left-right side distribution within each group (post hoc one-way ANOVA between left (L) and right (R) within each group, all p>0.05), b) cranio-caudal distribution regardless of group (post hoc one-way multiple ANOVA p<0.05), mean indicated by line. (TIF) [file pone.0240753.s001.tif]

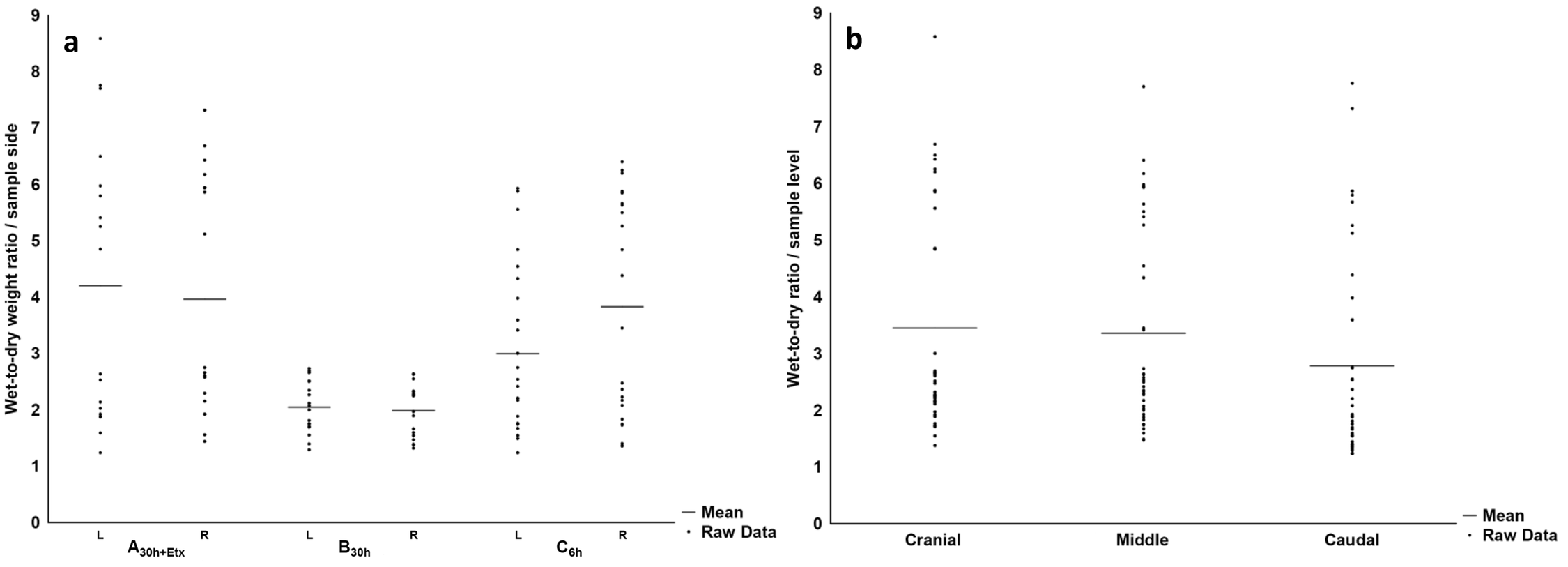

Supplement: S2 Fig — Raw data presentations of all wet-to-dry measurements in the experiment based on a) left-right side distribution within each group (post hoc one-way ANOVA between left (L) and right (R) within each group, all p>0.05), b) cranio-caudal distribution regardless of group (post hoc ANOVA p 0.23), mean indicated by line. (TIF) [file pone.0240753.s002.tif]

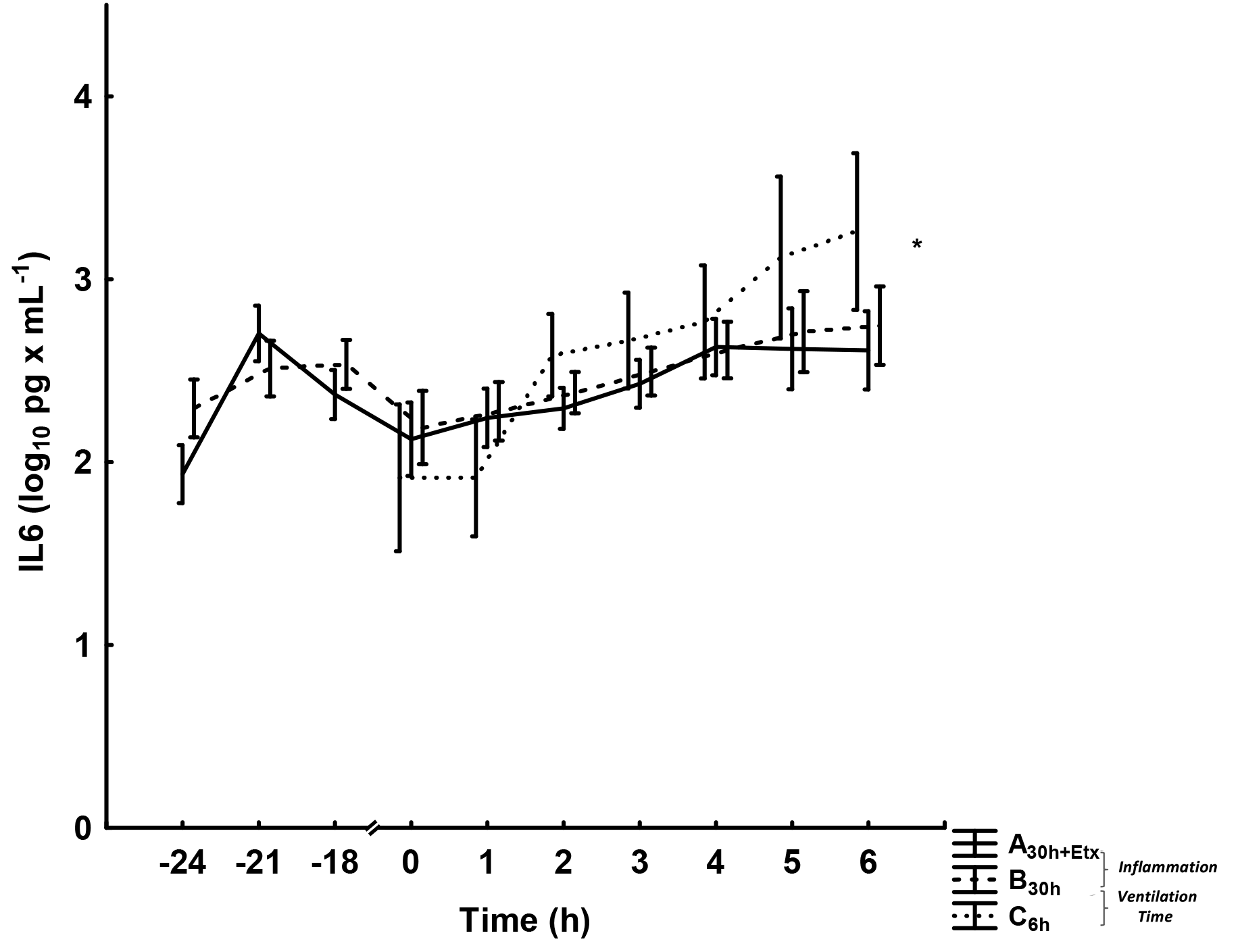

Supplement: S3 Fig — Mean±SEM, total group difference calculated between 0–6 h with ANOVA for repeated measures. Experimental parts Inflammation A30h+Etx vs. B30h (group p 0.84, group*time 0.49), Ventilation Time B30h vs. C6h (group p 0.63, group*time p<0.05*), axis scale changes at 0 hours. Post hoc statistics (group*time) present differing dynamics in cytokine escalation in C6h from the bacterial challenge 0–6 h. (TIF) [file pone.0240753.s003.tif]
