# Supplementary material for: Pre-exposure to mechanical ventilation and endotoxemia increases Pseudomonas aeruginosa growth in lung tissue during experimental porcine pneumonia
Source: PLoS One. 2020 Oct 27;15(10):e0240753. doi: 10.1371/journal.pone.0240753 (PMC7591049; doi:10.1371/journal.pone.0240753)
Supplement: S1 Table — Pseudomonas (P.) aeruginosa, bronchoalveolar lavage (BAL), colony forming unit (CFU), TNF-α (tumor necrosis factor alpha), IL6 (interleukin 6) at the start of the experiment -24 h (A30h+Etx and B30h), at the bacterial inoculation 0 h and at the end of the experiment 6 h, median(LQ/HQ), p upper refers to A30h+Etx vs. B30h, lower to B30h vs. C6h. (DOCX) [file pone.0240753.s004.docx]

**Supplementary Table 1** *Pseudomonas aeruginosa* and cytokines in bronchoalveolar lavages – complementary *post hoc* tests at – 24 and 0 h.

| **Variable** | **Group** | **-24 h** | **p** | **0 h** | **p** | **6 h** | **p** |
| --- | --- | --- | --- | --- | --- | --- | --- |
| ***P. aeruginosa* BAL** | A_30h+Etx_ | 0.0(0.0/0.0) |  | 0.0(0.0/0.0) |  | 4.0(3.8/4.0) |  |
| (log_10_CFU x 100µL^-1^) | B_30h_ | 0.0(0.0/0.0) | N/A | 0.0(0.0/0.0) | N/A | 4.4(4.1/5.0) | 0.24 |
|  | C_6h_ |  |  | 0.0(0.0/0.0) | N/A | 3.8(3.3/4.6) | 0.40 |
| **TNF-α BAL** | A_30h+Etx_ | 1.6(1.0/2.3) |  | 2.2(2.1/3.0) |  | 3.5(2.6/3.5) |  |
| (log_10_ng x L^-1^) | B_30h_ | 1.5(1.2/1.7) | 0.29 | 1.8(1.6/3.0) | 0.78 | 3.4(2.7/3.5) | 0.93 |
|  | C_6h_ |  |  | 1.8(1.7/1.9) | 0.84 | 3.3(2.8/3.6) | 0.75 |
| **IL6 BAL** | A_30h+Etx_ | 2.8(1.7/2.9) |  | 2.1(1.7/2.9) |  | 2.3(1.7/2.9) |  |
| (log_10_ng x L^-1^) | B_30h_ | 2.1(2.0/2.6) | 0.37 | 2.9(2.7/3.1) | 0.47 | 2.8(2.3/3.1) | 0.58 |
|  | C_6h_ |  |  | 2.6(1.7/2.9) | 0.37 | 2.6(2.0/3.0) | 0.65 |

*Pseudomonas* (*P.*) *aeruginosa,* bronchoalveolar lavage (BAL), colony forming unit (CFU), TNF-α (tumor necrosis factor alpha), IL6 (interleukin 6) at the start of the experiment -24 h (A_30h+Etx_ and B_30h_), at the bacterial inoculation 0 h and at the end of the experiment 6 h, median(LQ/HQ), p upper refers to A_30h+Etx_ vs. B_30h_, lower to B_30h_ vs. C_6h_.
