# Supplementary material for: Pre-exposure to mechanical ventilation and endotoxemia increases Pseudomonas aeruginosa growth in lung tissue during experimental porcine pneumonia
Source: PLoS One. 2020 Oct 27;15(10):e0240753. doi: 10.1371/journal.pone.0240753 (PMC7591049; doi:10.1371/journal.pone.0240753)
Supplement: S2 Table — Sham 30h (S30h, n = 1), Sham 6h (S6h, n = 2), heart rate (HR), mean arterial pressure (MAP), mean pulmonary arterial pressure (MPAP), cardiac index (CI), pulmonary capillary wedge pressure (PCWP), pressure (P), wet-to-dry ratio (WD), tumor necrosis factor alpha (TNF-α), interleukin (IL). Sham 30 h (S30, n = 1) absolute value, sham 6 h (S6, n = 2), mean±SD, median (LQ/HQ) as presented in the main manuscript. (DOCX) [file pone.0240753.s005.docx]

**Supplementary Table 2** Physiologic and laboratory variables in the sham animals

| **Variable** | **Group** | **-24** | **0** | **3** | **6** |
| --- | --- | --- | --- | --- | --- |
| **HR** | S_30h_ | 69 | 88 | 120 | 78 |
| (beats x min^-1^) | S_6h_ |  | 100±21 | 103±9 | 113±8 |
| **MAP** | S_30h_ | 94 | 94 | 114 | 92 |
| (mmHg) | S_6h_ |  | 84±14 | 86±4 | 81±8 |
| **MPAP** | S_30h_ | 21 | 21 | 27 | 24 |
| (mmHg) | S_6h_ |  | 18±1 | 21±1 | 21±1 |
| **CI** | S_30h_ | 2.1 | 3.2 | 4.8 | 2.7 |
| (L x min^-1^ x m^-2^) | S_6h_ |  | 3.3±0.3 | 3.2±0.5 | 3.6±1.1 |
| **PCWP** | S_30h_ | 12 | 13 | 10 | 15 |
| (mmHg) | S_6h_ |  | 8±1 | 9±0 | 8±0 |
| **Temp** | S_30h_ | 38.5 | 40.3 | 40.3 | 40.8 |
| (^∘^C) | S_6h_ |  | 39.3±0.5 | 39.8±1.4 | 40.3±1.1 |
| **P peak** | S_30h_ | 14 | 17 | 16 | 16 |
| (cmH_2_O) | S_6h_ |  | 15±2 | 16±4 | 17±6 |
| **P pause** | S_30h_ | 13 | 15 | 14 | 15 |
| (cmH_2_O) | S_6h_ |  | 14±2 | 15±4 | 16±7 |
| **P mean** | S_30h_ | 7 | 8 | 8 | 8 |
| (cmH_2_O) | S_6h_ |  | 8±1 | 8±1 | 9±3 |
| **PaO_2_/FiO_2_** | S_30h_ | 490 | 413 | 391 | 284 |
| (mmHg) | S_6h_ |  | 429±34 | 366±12 | 360±11 |
| **W/D** | S_30h_ |  |  |  | 1.8(1.7/2.3) |
|  | S_6h_ |  |  |  | 1.8(1.6/2.0) |
| **TNFα plasma** | S_30h_ | 2.2 | 2.2 | 2.5 | 2.3 |
| (log_10_ ng x L^-1^) | S_6h_ |  | 2.0±0.5 | 2.2±0.0 | 2.1±0.1 |
| **IL6 plasma** | S_30h_ | - | 2.0 | 2.4 | 2.1 |
| (log_10_ ng x L^-1^) | S_6h_ |  | 1.6±0.0 | 2.0±0.0 | 1.7±0.3 |
| **Leukocytes** | S_30h_ | 15 | 17 | 17 | 16 |
| (10^9^ x L^-1^) | S_6h_ |  | 19±8 | 21±7 | 22±7 |
| **Neutrophils** | S_30h_ | 8 | 9 | 11 | 10 |
| (10^9^ x L^-1^) | S_6h_ |  | 7 | 10 | 11 |
| **Platelets** | S_30h_ | 144 | 162 | 172 | 128 |
| (10^9^ x L^-1^) | S_6h_ |  | 393±115 | 356±106 | 337±115 |
| **Nitrite in urine** | S_30h_ | 4820 | 840 | 2260 | 2000 |
| (µmol x L^-1^)  mol x L^-1^) | S_6h_ |  | 3650(3480/3820) | 2920(1800/4040)0) | 3760(3160/4360)0) |
| **Lactate** | S_30h_ | 2.0 | 1.0 | 1.0 | 1.4 |
| (mmol x L^-1^) | S_6h_ |  | 2.1±0.5 | 1.0±0.1 | 0.9±0.1 |

Sham 30h (S_30h_, n=1), Sham 6h (S_6h_, n=2), heart rate (HR), mean arterial pressure (MAP), mean pulmonary arterial pressure (MPAP), cardiac index (CI), pulmonary capillary wedge pressure (PCWP), pressure (P), wet-to-dry ratio (WD), tumor necrosis factor alpha (TNF-α), interleukin (IL). Sham 30 h (S30, n=1) absolute value, sham 6 h (S6, n=2), mean±SD, median (lower/upper quartile) as presented in the main manuscript.
