# Supplementary material for: Pre-exposure to mechanical ventilation and endotoxemia increases Pseudomonas aeruginosa growth in lung tissue during experimental porcine pneumonia
Source: PLoS One. 2020 Oct 27;15(10):e0240753. doi: 10.1371/journal.pone.0240753 (PMC7591049; doi:10.1371/journal.pone.0240753)
Supplement: S1 File — (PDF) [file pone.0240753.s007.pdf]

To the Editor of PLoS ONE

Dear Sir,

In my capacity as Senior Statistician at the Centre for Clinical Research Sörmland, County Council Region Sörmland, I was asked by the authors, Sperber J et al., of the manuscript " **PONE-D-20-18632 Pre-exposure to mechanical ventilation and endotoxemia additively increases Pseudomonas aeruginosa growth in lung tissue during experimental porcine pneumonia**" to respond to the Editor´s comment below. I have reviewed the statistical part and calculations in the manuscript and formulated a response following below.

Editorial comment: "*In the statistics part you state "Comparative group statistics in the experimental parts Inflammation (A30h+Etx vs. B30h) and Ventilation Time (B30hvs. C6h) were based on data from the last 6 h of the experiment (the bacterial phase). No multigroup comparisons including all three groups were used in the experiment." However, you used group B30h twice in your statitics, which means that you apparently treated the setting as two independent experiments. This is clearly not the case. Although being no biostatistician, I think you have to adjust the level of significanse to the fact that you used group B30h twice.*"

My response: I understand the comment. I see two parts of the comment. 1) Are the experiments independent or dependent? 2) Is a correction of the level of significance warranted?. These two comments are not absolutely causal. If experiments are dependent, other statistical methods are warranted. Corrections to the level of significance is warranted in the case of multiple comparisons are made.
In my mind, the manuscript is constructed as two different main aims, investigated in two different experiments. In the first experiment group A was compared with group B and in the second experiment group B was compared with group C. Thus, three independent groups of animals were involved in two independent experiments. Statistically, these experiments are independent, and the statistical methods used in the manuscript are adequate. As to the question of correction of the level of significance, as this is an exploratory investigations with variables that biologically correlate to each other, I do not find that a correction of the level of significance is warranted.
In conclusion I find the statistical methods used by the authors to be adequate.

Eskilstuna the 20th of August 2020,

Nicklas Pihlström
Senior Statistician
Telephone: +46 105226
nicklas.pihlstrom@regionsormland.se
