# Supplementary material for: Pre-exposure to mechanical ventilation and endotoxemia increases Pseudomonas aeruginosa growth in lung tissue during experimental porcine pneumonia
Source: PLoS One. 2020 Oct 27;15(10):e0240753. doi: 10.1371/journal.pone.0240753 (PMC7591049; doi:10.1371/journal.pone.0240753)
Supplement: S2 File — (DOCX) [file pone.0240753.s008.docx]

**Methods, supplementary information**

**Anesthesia and surgical procedure**

The time from induction of anesthesia until the experimental starting point was approximately one hour in all animals and included the preparatory surgery and the preparation of the bacterial inoculum (Figure 1 in main document). The pigs were sedated with tiletamin 3 milligrams (mg) x kilogram (kg)^-1^, zolazepam 3 mg x kg^-1^, and xylacin 2.2 mg x kg^-1^. Morphine 20 mg and ketamine 100 mg were given in an auricular vein. Anesthesia was maintained with pentobarbital 8 mg x kg^-1^ x h^-1^ and morphine 0.26 mg x kg^-1^ x h^-1^. To facilitate ventilator management and counteract shivering and coughing muscle relaxation was maintained with an infusion of rocuronium at an initial rate of 2 mg x kg^-1^ x h^-1^. Tracheal intubation was performed via tracheostomy. The right side of the neck was soap washed, disinfected with alcohol and dressed in sterile drapes. A 10-centimeter (cm) incision preceded blunt dissection of the neck vessels. A 5-French (F) arterial catheter was inserted in the Thyrocervical trunk. A central venous catheter and a Swan-Ganz pulmonary artery catheter were introduced via the external jugular vein. The left subcostal area of the abdomen was soap washed, disinfected with alcohol and dressed in sterile drapes. A 15-cm left sided subcostal incision was followed by blunt dissection of the muscle layers and opening of the peritoneum. The spleen could be eviscerated and the splenic hilus identified. A guide wire was introduced into the splenic vein whereupon a 5F catheter was inserted 15 cm to reach a tip location in the portal vein. The portal catheter was placed primarily to harmonize for comparability the current experiment with preceding experiments [1, 2]. A surgical cystostomy catheter was inserted after a small midline incision. To reduce the risk of bacterial contamination of the model cefuroxime 750 mg was administered after the surgical preparations. Endotoxin 0.063 *µ*g x kg^-1^ x h^-1^ was administered via the auricular vein for 24 hours in one group, A_30h+Etx_.

Before the start of the study protocol, a suction catheter was inserted blindly into the tracheal tube until it reached mechanical resistance. After bronchoalevolar lavage (BAL) for culture and laboratory analyses the bacterial suspension was injected blindly into the lungs via the same catheter. A recruitment maneuver was performed with a plateau pressure of 30 cm H_2_O for 10 seconds (s). The inoculation of *P. aeruginosa* denoted the start of the experiment. The animals were kept in a supine position throughout the bacterial part of the experiment.

**Bacterial inoculum**

The aim of the bacterial preparation was to produce a 20 mL bolus consisting of 1x10^11^ colony forming units (cfu) of *P. aeruginosa* (5 x 10^9^ cfu x mL^-1^). The strain was isolated from a previous porcine experiment and naturally resistant to cefuroxime. It was O-antigen serotyped to O3 by a slide agglutination test with commercial antisera (Bio-Rad Laboratories AB, Solna, Sweden) at the section for Clinical Microbiology and Infectious Medicine (Uppsala, Sweden). Bacteria from overnight cultures on Cystine-Lactose-Electrolyte-Deficient (CLED) agar (BD Diagnostics, Stockholm, Sweden) were dissolved in lysogeny broth (LB) according to Miller [3] (VWR, Leuven, Belgium). The optical density of the bacterial solution was measured with light absorbance spectrophotometry at a wavelength of 595 nanometers; a target value of 0.7 was reached by either dilution of the suspension or addition of more bacteria. One hundred mL of the final suspension were further diluted with another 100 mL of LB and incubated at 37° C for 60 minutes (min). The incubated solution was centrifuged for 10 min at 20° C to form bacterial pellets that were dissolved in 20 mL of sodium chloride 0.9%. One hundred µL were diluted 1:10^7^ to confirm the bacterial concentration of the bolus dose by culture on CLED agar.

**Protocol**

The initial respirator settings (Servo i, Siemens Elema, Stockholm, Sweden) in all groups were volume control mode, tidal volume 10 mL x kg^-1^, PEEP 5 cmH_2_O and rate 25 breaths x min^-1^. Initial inspired fraction of oxygen was 0.3 for all animals. One animal in the 30-hour experiment and two in the six-hour experiment served as sham controls. The sham animals were not given endotoxin or a bacterial challenge but were in all other respects treated according to the protocol. Adjustments were made every hour to reach normal ventilation, defined as arterial partial pressure of carbon dioxide between 4.5 and 6.5 kPa by increments or decrements of 10% of respiratory rate. Arterial partial pressure of oxygen (PaO_2_) was kept between 10 and 18 kPa by either an increment of 10% or a decrement of 5% of inspired fraction of O_2_ (FiO_2_). If mean arterial pressure (MAP) equaled mean pulmonary arterial pressure (MPAP), due to the initial pulmonary hypertension seen in endotoxemic pigs [4], within the first 90 min of the experiment, norepinephrine was given in boluses of 40 µg. If MAP equaled MPAP after 90 min, Ringer´s acetate was given in bolus doses of 15 mL x kg^-1^ maximum twice, and a norepinephrine bolus of 20 µg was given followed by a norepinephrine infusion of 20 µg x mL^-1^ with a starting rate of 5 mL x h^-1^. If MAP regardless of MPAP were lower than 60 mmHg after 90 min or if cardiac index (CI) were below 2 L x min^-1^ x m^-2^, a norepinephrine infusion of 20 µg x mL^-1^ was started with an initial rate of 5 mL x h^-1^ without a preceding bolus. The infusion dose was doubled every time MAP or CI relapsed below the preset limits.

**Interventions**

Initially, tidal volume was 10 mL x kg^-1^, PEEP 5 cm H_2_O, respiratory rate 25 x min^-1^ and inspired oxygen fraction (FiO_2_) 0.3. The respiration was adjusted to meet an arterial partial pressure of carbon dioxide (PaCO_2_) value from 4.5-6.5 kilo pascal (kPa) by an increment or decrement in the respiratory frequency of 10%. Predefined increments of FiO_2_ (0.3-0.6-0.8-1.0) were performed at arterial partial pressure of oxygen (PaO_2_) values below 10 kPa simultaneously with changes in predefined PEEP levels (5-8-10-14 cm H_2_O). If the plateau pressure were over 30 cm H_2_O, the tidal volume was reduced to 7 mL x kg^-1^ and the inspiratory to expiratory ratio was changed from 1:2 to 1:1. The alveolar recruitment maneuver (ARM) consisted of stepwise increments of PEEP until the peak pressure reached 35 cm H_2_O, followed by prolonged inspiration for 10 seconds (s). ARM was performed at the start of the protocol (-24 h) and after each change of position in the 30 h groups, as well as after bacterial inoculation at 0 h in all groups. During the first 90 minutes (min) of the experiment, norepinephrine was used in boluses of 40 µg if mean arterial pressure (MAP) equaled mean pulmonary arterial pressure (MPAP). A MAP value, regardless of MPAP, that was below 60 mmHg after 90 min was treated with a bolus of Ringer´s acetate 15 mL x kg^-1^, a 1 mL bolus of norepinephrine 20 µg x mL^-1^, followed by a norepinephrine infusion of the same concentration starting at 5 mL x h^-1^. At relapse of MAP below 60 mmHg, the infusion dose was doubled.

**Measurements**

At the experimental endpoint at 6 h, the animals were euthanized by potassium injection and the sternum was opened. Lung tissue bacterial cultures and weight measurements were based on three dorsal samples from the right lung cranial, middle and caudal lobes, as well as three corresponding level samples from the left lung. Approximately 1 gram (g) from each sample was used for bacterial cultures. Three mL of sodium chloride 0.9% were added, followed by 4 min of mechanical homogenization with a Stomacher 80 Biomaster (Seward, Worthing, UK). One hundred µL were sequentially diluted until 1:10^4^ and cultured in a single repetition on CLED agar plates over night at 37 ºC. The numbers of cfus from the countable plates were converted to units x g^-1^ lung tissue. The remainders of the six samples, ranging from 10-40 g, from each animal were weighed directly and after drying for 12 h at 60 ºC. Bronchoalveolar lavages, performed by method of blind bronchial sampling with 20 mL of saline 0.9%, for cultures and cytokine measurements were performed at 0 h and before the end of the experiment at 6 h.

Physiological variables (i.e. respiratory, circulatory, temperature and diuresis) were measured hourly and blood samples were taken from the artery for inflammatory markers and blood gas analysis. Blood gas analyses from the portal vein were taken at 0, 3 and 6 h. The blood samples were centrifuged to retain plasma which was frozen at -18 ºC for later analysis. Commercial porcine-specific sandwich enzyme-linked immunosorbent assay (ELISA) was used for the determination of tumor necrosis factor alpha (TNFα) and interleukin 6 (IL6) in plasma and in BAL (DY690B (TNFα) and DY686 (IL6), R&D Systems, Minneapolis, MN, USA). The ELISAs had intra-assay coefficients of variation (CV) of less than 5% and total CV of less than 10%. All baseline measurements and sampling procedures were performed before the instillation of bacteria at 0 h.

**Statistics**

The animals were allocated to groups of 6-8 animals by block randomization simultaneously with the previously published experiment [5]. Comparative group statistics in the experimental parts *Inflammation* and *Ventilation Time* were based on data from the last 6 h of the experiment (the bacterial phase). A general linear model (GLM) was used for group comparisons in the lung tissue sample variables (i.e. bacterial growth and wet-to-dry weight ratio). Random effects were introduced into the model to account for the within-subject dependencies of the six simultaneous lung tissue samples from each individual, making the GLM a mixed model. Because the bacterial inoculum was delivered blindly to either the right or left lung, the tissue samples in each animal were statistically analyzed using three levels (cranial-middle-caudal) consisting of the right and left corresponding samples. Repeated measures were analyzed with analysis of variance (ANOVA) for repeated measures. Only the group factor is presented as a p-value in the results from either the GLM or ANOVA for repeated measures. Inoculated dose and bacterial counts in BAL were analyzed with Mann-Whitney U-test for each experimental part (*Inflammation* and *Ventilation Time*) based on non-normal distribution, but data was still presented in a logarithmic form for coherence within the presentation. In analogy with earlier publications all cytokines were logarithmically transformed based on log-normal distribution [1, 2, 5]. Statistica^TM^ (Statsoft, Tulsa, OK, version 13) was used for the statistical calculations and control of relevant assumptions. A p-value of < 0.05 was considered significant. A senior statistician approved the statistical design. Sham animals are only presented in the supplementary material as descriptive data.

No power calculation was conducted for this specific experiment since we had no previous data on bacterial behavior in our models. Instead, we used the power calculation for the preceding inflammatory experiments [1, 2, 5]. It was based on a systemic TNF-alpha difference of 15% at 6 hours, an alpha error of 0,05, a power of 0,8, and an SD of 10%, which yielded six evaluable animals per group. The choice of 8 animals per group in the previously published day-based experiment [5] was based on this calculation while allowing for a slightly larger variability in the bacterial outcome variable. As we started with the day-based experiment we could appreciate the bacterial growth in lung tissue better. Based on this data we reduced the number of animals in the 30 h experiments, which were completed at the end of the experimental period, from eight to six to meet the 3R principle. In summary, we reduced the number of animals as we believed we could meet the required difference in the main outcome variable anyway.

The rational for the use of a general linear model in the lung tissue variables (bacterial growth and wet-to-dry ratio) was that each animal yielded six internally dependent samples that were taken simultaneously. Based on the size of the experimental animal we could not use the whole lung to prepare one measure per total lung weight – which is possible in smaller animals such as mice. Additionally, the bacterial growth was significantly differing in the lung parenchyma in cranio-caudal direction, with the highest bacterial count in caudal and the lowest in cranial samples. This presentation was most pronounced in two groups, B_30_ and C_6h_, and not as much in group A_30h+Etx_ that displayed a more homogenous growth in all three levels. We could not see differences between sides (right vs left lung) although the inoculated dose was deemed to have been deposited to either the right or the left lung - based on the length of the suction catheter used for blind bronchial sampling and inoculation at the start of the experiment. The level values were more similar and made it possible to group the results into the subcategory “level”, each made up of two individual samples (right and left). The two samples from each level in each animal were handled in the statistical analysis as separate values and not as composites or means. **Supplementary Figure 1** describe the raw data distribution of colony forming units of all samples in the experiment in the left-to-right and cranial-to-caudal directions. *Post-hoc* tests of all samples show no difference between sides (univariate ANOVA p>0.05), but a significant difference in cranio-caudal direction (multiple ANOVA p<0.05). The same raw data set-up is presented for all wet-dry measurements in the experiment with no significant differences in either left-right or cranio-caudal direction (**Supplementary Figure 2**).

As a total the logarithmic growth (and WD) values of all samples in the experiment fulfilled the criteria for normality. Normality of data was evaluated in the same way for all variables in the experiment – a histogram of raw residuals for normality distribution, a normal probability vs. raw residuals plot for linearity and a scatterplot for homoscedasticy.

*
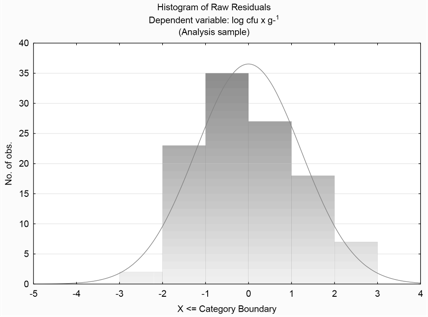

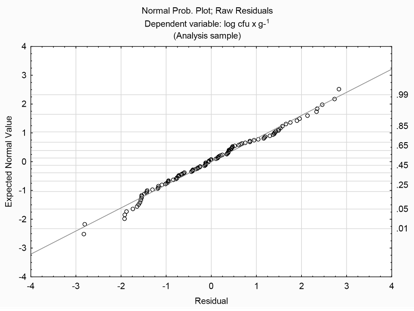

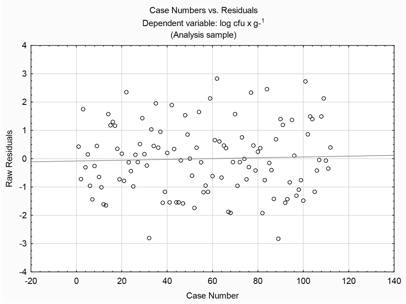
*

Hence, a model was needed that could accommodate both internally dependent values and differences within each individual that could not be made into a useful mean value. The use of a GLM with random factor in the level was an option that could meet these requirements. The group was the fixed factor and the level was named the random factor. The resulting equation (group, level (r), group*level (r)) was very similar to the ANOVA for repeated measures used in the longitudinal repeated measure variables (group, time, group*time). It serves to say that we could have used location (all six samples) instead of level with the same result. In the previously published article [5] we presented a graph with bacterial growth based on the three levels why the same approach was used in the current experiment.

If the growth values were similarly distributed within the lungs, it would be appropriate to make one mean value for each individual and perform statistics on the group mean. As this prerequisite was not present mean value formation in each individual was deemed inappropriate in this experimental set up. Additionally, the internal samples were not independent from each other. The random factor was introduced into the level-factor both to account for the internal dependencies of the samples and the inherent randomness of the actual sampling in the lung tissue even if three gross levels in each animal was the intention.

**References**

1. Sperber J, Lipcsey M, Larsson A, et al (2013) Lung Protective Ventilation Induces Immunotolerance and Nitric Oxide Metabolites in Porcine Experimental Postoperative Sepsis. PLoS ONE 8:e83182. https://doi.org/10.1371/journal.pone.0083182

2. Sperber J, Lipcsey M, Larsson A, et al (2015) Evaluating the effects of protective ventilation on organ-specific cytokine production in porcine experimental postoperative sepsis. BMC Pulm Med 15:. https://doi.org/10.1186/s12890-015-0052-9

3. Miller JH (1972) Experiments in molecular genetics. Cold Spring Harbor Laboratory, Cold Spring Harbor, N.Y.

4. Schmidhammer R, Wassermann E, Germann P, et al (2006) Infusion of increasing doses of endotoxin induces progressive acute lung injury but prevents early pulmonary hypertension in pigs. Shock Augusta Ga 25:389–394. https://doi.org/10.1097/01.shk.0000209529.43367.00

5. Sperber J, Nyberg A, Lipcsey M, et al (2017) Protective ventilation reduces Pseudomonas aeruginosa growth in lung tissue in a porcine pneumonia model. Intensive Care Med Exp 5:40. https://doi.org/10.1186/s40635-017-0152-3
